# Micro and Macro Flooding Mechanism and Law of a Gel Particle System in Strong Heterogeneous Reservoirs

**DOI:** 10.3390/gels10020151

**Published:** 2024-02-19

**Authors:** Rongjun Ye, Lei Wang, Wenjun Xu, Jianpeng Zhang, Zhengbang Chen

**Affiliations:** 1 School of Petroleum Engineering, Yangtze University, Wuhan 430100, China; xuwenjun@yangtzeu.edu.cn (W.X.); jianpzhang@163.com (J.Z.); chen03012024@163.com (Z.C.); 2 Key Laboratory of Drilling and Production Engineering for Oil and Gas, Wuhan 430100, China

**Keywords:** strong heterogeneous reservoir, channeling path, gel particle, physical simulation, enhanced oil recovery (EOR)

## Abstract

To address the issue of ineffective injection resulting from the consistent channeling of injected water through highly permeable channels in ultra-deep, high-temperature, high-salinity, and strongly heterogeneous reservoirs during the production process, a gel particle profile control agent suitable for high-temperature and high-salinity conditions was chosen. With the help of the glass etching visual microscopic model and the heterogeneous long core model, the formation mechanism of a water flooding channeling path and the distribution law of the remaining oil were explored, the microscopic profile control mechanism of the different parameters was clarified, and the profile control effect of macroscopic core displacement was analyzed. The research shows that the formation mechanism of a water flooding channeling path is dominated by the distribution law of the permeability section and the connection mode between different penetration zones. The remaining oil types after water flooding are mainly contiguous block, parallel throats, and multi-branch clusters. The profile control effect of gel particles on reservoir vertical heterogeneity is better than that of reservoir lateral heterogeneity. It was found that 10 wt% submicron particles with a median diameter of 600 nm play a good role in profiling and plugging pores of 5–20 μm. In addition, 10 wt% micron-sized particles with a median diameter of 2.63 μm mainly play a strong plugging role in the pores of 20–30 μm, and 5 wt% micron-sized particles with a median diameter of 2.63 μm mainly form a weak plugging effect on the pores of 10–20 μm. The overall profile control effect of 10 wt% submicro particles is the best, and changes in concentration parameters have a more significant effect on the profile control effect. In the macroscopic core profile control, enhanced oil recovery (EOR) can reach 16%, and the gel particles show plugging, deformation migration, and re-plugging. The research results provide theoretical guidance for tapping the potential of the remaining oil in strong heterogeneous reservoirs. To date, the gel particles have been applied in the Tahe oilfield and have produced an obvious profile control effect; the oil production has risen to the highest value of 26.4 t/d, and the comprehensive water content has fallen to the lowest percentage of 32.1%.

## 1. Introduction

With the development of China’s oilfields gradually entering the middle and late stages, the long-term water injection development method has led to a significant increase in the water content of the produced liquid of oil wells. For heterogeneous strong reservoirs, this will further enhance their heterogeneity, resulting in excess dispersion of the remaining oil. Therefore, the remaining oil in a strong heterogeneous reservoir developed by long-term water injection is rich in potential space [1,2,3,4]. The reservoir which is the fourth Triassic member in the T block of the Tahe oilfield is an ultra-deep, high-temperature, and high-salinity reservoir [5,6]. There are two sets of interlayers with moderate heterogeneity in the longitudinal direction and strong heterogeneity in the plane. After long-term water injection development, the current oil recovery is still low. The main reason for this is that there is a local concentration of plane streamlines in the water injection block, and the injected water is easy to channel along the dominant channel, resulting in premature water breakthrough in the oil well and ineffective water injection. Behzad Vaferi et al. applied deep-learning alternative models to prove that the water injection pattern strongly affects the water channeling reduction and the ultimate oil recovery increase [7]. However, at present, studies on the formation law of micro-waterflood channels in real-model experiments are still insufficient, and it is difficult to take effective measures to deal with the problems in water channels [8,9].

As an important measure to improve the development effect of water flooding, control water, and stabilize oil, profile control and flooding technology has been widely used in oilfields at home and abroad [10,11]. The essence of this technology is to improve reservoir heterogeneity and improve the water flooding sweep efficiency and oil washing efficiency by injecting chemical agents with both profile control abilities and oil washing abilities into injection wells, so as to achieve the purpose of enhance oil recovery (EOR) [12,13]. For the harsh conditions of ultra-deep, high-temperature, and high-salt reservoirs, conventional profile control and flooding systems such as inorganic gel have good temperature and salt resistance but short gelation times, and it is difficult to control the gelation time [14,15]. Inorganic particles are less affected by temperature and salinity but are poor in selective plugging, and raw materials are greatly affected by the origin [16,17,18]. Foams have strong selective plugging abilities which enables them achieve high-temperature and high-salt resistance, but they have short validity periods and involve complex construction technology [19,20,21,22]. Resin has a high plugging strength and a long validity period but poor selective plugging and is difficult to remove after plugging [23,24,25]. Organic polymer particles are widely used because of their low cost, good selectivity, and ability to absorb water and expand to form deep profile control under high-temperature and high-salt conditions. At present, research into organic particles is relatively mature, which includes research regarding profile control colloidal dispersion gel (CDG), precrosslinked gel particles (PPGs), polymer microspheres, and gelled dispersion particles (DPGs) [26,27,28,29,30]. Considering the technical comparison above and factors such as temperature and salt resistance, deep migration, strong reservoir matching, and simple construction, the organic polymer particle control agent is still the best choice for the control of strong heterogeneous reservoirs.

In recent years, a kind of granular micro and nano expanded polymer gel micellar emulsion technology has been developed. The preparation process of this technology is simple, efficient, and environmentally friendly, and the particles at the nanometer to millimeter level can be prepared according to the requirements of the reservoir. However, the influence of the key injection parameters of the particles on the oil displacement effect is still unknown. Therefore, on the basis of this technology, this paper developed a kind of reservoir pore throat size particle suitable for block T in the Tahe oilfield. At the microscopic level, the visual microscopic model of glass etching was used to compare and analyze the oil displacement effect of the system under different key parameters, and the formation mechanism of a water drive channel and the distribution law of remaining oil were studied. On the macro level, the core displacement experiment was used to analyze the displacement effect of gel particles and verify the characteristics of the micro displacement mechanism.

## 2. Results and Discussion

### 2.1. Study on Water Flooding Microscopic Channeling Mechanism and Remaining Oil Distribution Law

#### 2.1.1. Mechanism Analysis of Water Flooding Channeling Path

**Figure 1:**
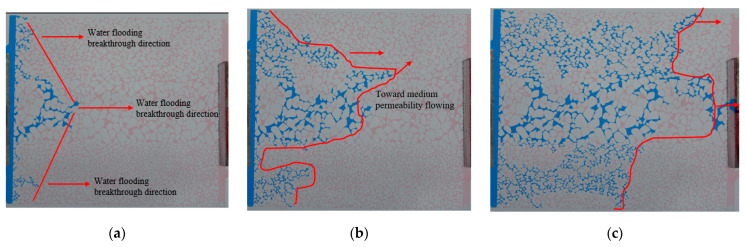
Water flooding phenomena in different periods: (**a**) Initial stage of water flooding; (**b**) Middle stage of water flooding; (**c**) Later stage of water flooding.

**Figure 2:**
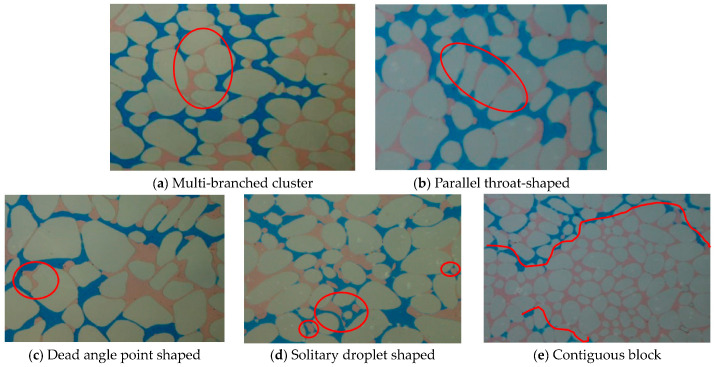
The microscopic local enlargement diagram of various remaining oil forms.

**Figure 3:**
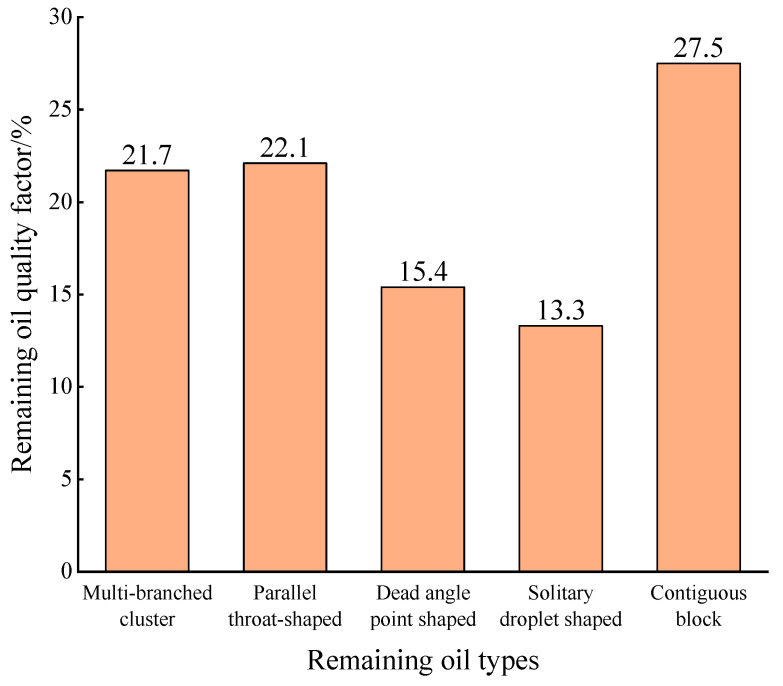
Pile-up histogram of various types of remaining oil after water flooding.

**Figure 4:**
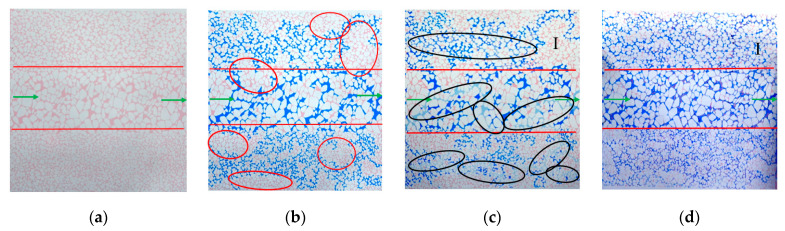
Experiment 1: Remaining oil distribution map (vertical, 10 wt% submicron scale 100 μL): (**a**) oil flooding; (**b**) water flooding; (**c**) particle flooding; (**d**) subsequent water flooding.

**Figure 5:**
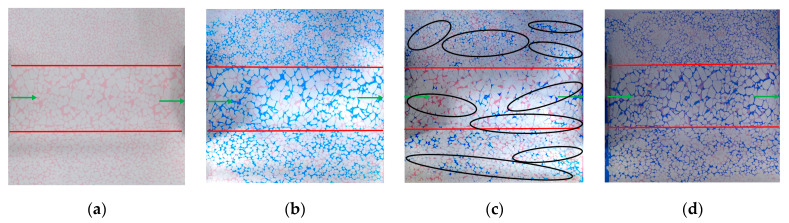
Experiment 1: Remaining oil distribution map (horizontal, 10 wt% submicron scale 100 μL): (**a**) oil flooding; (**b**) water flooding; (**c**) particle flooding; (**d**) subsequent water flooding.

**Figure 6:**
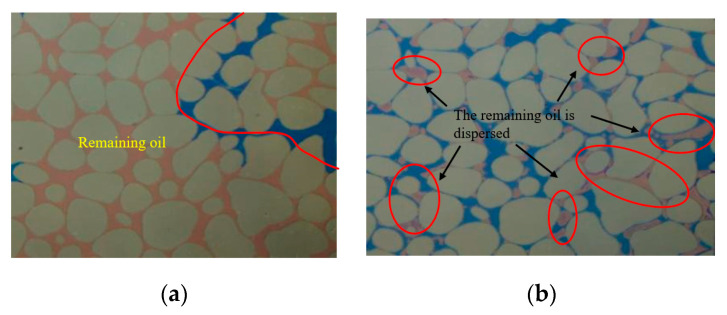
Experiment 1: Local enlargement of Region I: (**a**) particle flooding; (**b**) subsequent water flooding.

**Figure 7:**
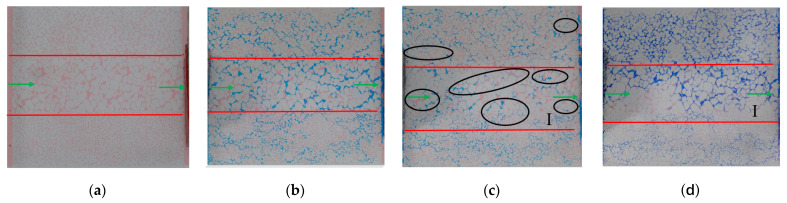
Experiment 3: Remaining oil distribution map (vertical, 10 wt% micron 100 μL): (**a**) oil flooding; (**b**) water flooding; (**c**) particle flooding; (**d**) subsequent water flooding.

**Figure 8:**
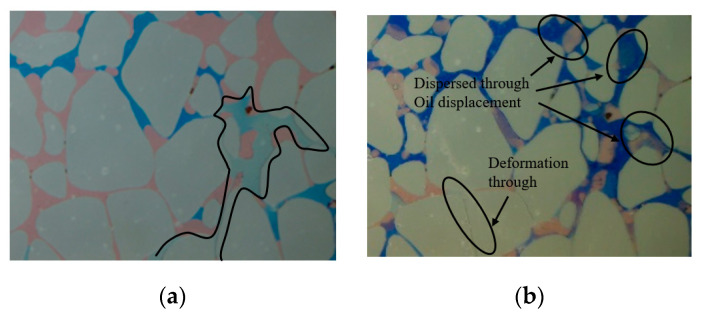
Experiment 3: Local enlargement of Region I: (**a**) particle flooding; (**b**) subsequent water flooding.

**Figure 9:**
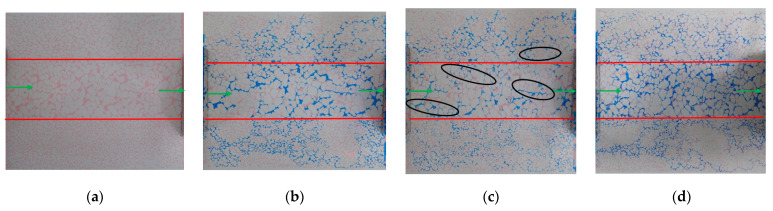
Experiment 4: Remaining oil distribution map (vertical, 5 wt% micron 100 μL): (**a**) oil flooding; (**b**) water flooding; (**c**) particle flooding; (**d**) subsequent water flooding.

The water flooding process is shown in Figure 1. In Figure 1, Figure 2, Figure 3, Figure 4, Figure 5, Figure 6, Figure 7, Figure 8 and Figure 9, blue is the simulated water, red is the simulated oil, white is the rock skeleton, and the displacement direction is from left to right. The red circle indicates remaining oil, the black circle represents gel particles, and the green arrow indicates the inlet and outlet. In the early stage of water flooding, the overall performance of the water drive front is finger breakthrough. This means that the high-permeability section advances rapidly, while the middle- and low-permeability sections on both sides progress slowly, leading to the formation of local finger phenomenon within each penetration section (Figure 1a). With the increase in displacement pressure and the difference of oil−water viscosity, the injected water not only advances forward, but also spreads to both sides of the model (Figure 1b). The analysis shows that the occurrence of this phenomenon depends on the degree of pore connectivity at the interface between the permeability zones. The connection of different permeability zones belongs to the intralayer connection, where the pores on both sides are in an almost interconnected state, with the large pores dominating. At this time, the degree of pore connection has improved, and the speed of the two sides is faster. If the connection belongs to the interlayer connection, meaning there is a certain degree of interlayer at the connection, which makes the displacement phase unable to spread to another permeability zone, then the degree of pore connection is poor, and the speed of the two sides is slow or almost zero. However, the formation of the overall fingering phenomenon of water flooding is still dominated by the pore size. The injected water is rapidly channeled along the middle high-permeability section to form the dominant channel, followed by the middle-permeability section above (Figure 1c).

#### 2.1.2. Remaining Oil Distribution

After water flooding, the oil−water distribution image of the model is locally enlarged, the distribution law of the remaining oil is analyzed, and the remaining oil is quantitatively assessed using image analysis software. As shown in Figure 2, the distribution types of the remaining oil in the model after water flooding are summarized as follows: (1) Large pores surrounded by multiple fine pore throats, making it difficult for injected water to overcome large seepage resistance and form multi-branch cluster residual oil (Figure 2a); (2) A small pore throat is formed in parallel with the large pore channel. The injected water tends to enter the larger pores, bypassing the smaller ones, and eventually establishes a consistent and stable flow pathway within the larger pores, resulting in the formation of parallel residual oil reservoirs. (Figure 2b); (3) In situations where there is only a single flow outlet outside the channel and the injected water flows vertically towards the outlet end, it becomes challenging for the injected water to alter the direction of its streamline and create a pressure differential within the channel. As a result, this leads to the formation of dead-angle points where residual oil accumulates. (Figure 2c); (4) When the rock surface is hydrophilic, the injected water tends to flow along the contours of the rock surface, thereby creating isolated droplets of residual oil in the central region of the larger channel. (Figure 2d); (5) In the place where a large number of small pores exists or the large pores are nearly enclosed by small pores, it becomes challenging for the injected water to create a local breakthrough and subsequently disperse the flake oil, thus forming a contiguous block of residual oil (Figure 2e) [31,32,33,34]. The distribution of various types of remaining oil after water flooding is statistically analyzed. It can be seen from Figure 3 that the contiguous block, parallel throat shape, and multi-branch cluster shape account for more than 20%, and the remaining oil types mainly consist of these three types.

### 2.2. Study on Micro Flooding Mechanism of Key Parameters of Gel Particles

In this study, we change the heterogeneous distribution form of the model, the size of the injected particles, and the concentration of the injected particles. We compare the microscopic profile control effects under different key parameters of the gel particle profile control agent.

#### 2.2.1. Heterogeneous Distribution Form of the Model

Changing the form of the heterogeneous distribution, the vertical placement represents vertical heterogeneity, while the horizontal placement represents plane heterogeneity. Comparing the experimental results of Experiment 1 and Experiment 2, as shown in Figure 4 and Figure 5, the colorless liquid area in the subsequent water flooding represents water after high-temperature fading. The red circle indicates oil, the black circle represents gel particles, and the green arrow indicates the inlet and outlet. Under the condition of longitudinal heterogeneity, the water flooding efficiency is highest in the permeability section. Simultaneously, the influence of gravity causes an excess accumulation of the remaining oil in the upper part of the permeable section. However, sizable pockets of remaining oil are still observed in the lowest part of the low-permeability section, highlighting that pore size remains a critical factor in the formation of residual oil. The type of residual oil after water flooding is mainly contiguous residual oil. A large number of unexpanded gel particles enter the hypertonic channel. After 72 h of thermal expansion of gel particles in the model, large pores can be effectively blocked. A large amount of contiguous residual oil is dispersed into residual droplets through subsequent water flooding and peristaltic gel particles, and then it flows out with the water [35,36,37] (Figure 6). The remaining oil distribution in the three permeable sections after water flooding under the plane heterogeneity is relatively uniform, mainly existing in parallel throats and multi-branch clusters. The presence of expansion particles enables the efficient utilization of residual oil in the three penetration sections. However, the effectiveness primarily depends on profile adjustments, and the oil displacement effect is not particularly significant. From an overall perspective of the profile control effect image, the gravitational impact of vertical heterogeneity affects the water flooding effect. When it comes to controlling displacement in a longitudinal heterogeneous reservoir, the impact of expansion particles surpasses that of a transverse heterogeneous reservoir. This observation is further supported by the analysis conducted on the enhanced oil recovery (EOR) below.

#### 2.2.2. Different Injection Particle Sizes

By changing the injection particle size, the experimental results of Experiment 1 and Experiment 3 are compared. Figure 4 and Figure 7 show that 10 wt% submicron particles (600 nm) can penetrate the middle/high/low-permeability sections. The subsequent water flooding can produce steering in these sections, leading to the effective utilization of the remaining oil. The 10 wt% micron particles (2.63 μm) primarily flow into the main channel (20–30 μm) of the high-permeability section, causing a significant plugging effect, while they hardly enter the medium- and low-permeability sections. Therefore, the subsequent water flooding mainly turns to the medium- and high-permeability remaining oil area and continues to advance along the larger pores in the low-permeability section. During subsequent water flooding process after 72 h of heat preservation, the particles disperse and deform from their original plugging position to other areas, creating an oil displacement effect. They deform through the fine pore throat to form re-displacement and plugging (Figure 8). From the pressure data in Table 1, it can be seen that the subsequent water flooding pressure in Experiment 1 and Experiment 3 is 0.11 MPa and 0.13 MPa, respectively. The better the pressure-lifting effect of large particles, the more effective the plugging effect, and the stronger the subsequent liquid flow steering ability. However, when combined with the experimental image comparison results mentioned above, it can be observed that its profile control effect in the low permeability section is inferior to that of small particles. It can be observed that increasing the size of injected particles can elevate the injection pressure and counteract the large capillary force of small pore throats. However, due to its large particle size, it fails to enter the large pore channels of the low-permeability section effectively. This limitation hinders the improvement the internal heterogeneity in the low permeability section, resulting in a poor outcome in enhancing oil recovery. Therefore, when selecting the injection particle size of the profile control agent, it is important not only to consider the pressurization effects but also to comprehensively consider the migration and plugging effect in different permeability sections.

#### 2.2.3. Different Injection Concentration

By changing the injection concentration, we compare the experimental results of Experiment 3 and Experiment 4. Figure 7 and Figure 9 show that 5 wt% micron particles (2.63 μm) mainly form plugging in the middle- and high-permeability sections. However, due to the low concentration of gel particles, the plugging strength is weak, so the subsequent profile control effect is worse than that of the 10 wt% micron (Figure 9d). Moreover, due to the decrease in concentration, the likelihood of collisions between particles is reduced, making it challenging for particles to adsorb and coalesce with each other. It is difficult to achieve a state of aggregation and accumulation to block the large pores, which hinders the increase in displacement pressure. The particle displacement pressure in Experiment 4 is equal to the initial water displacement pressure (Table 1). However, since the diameter of large-particle-size and low-concentration particle aggregates is smaller than that of large-particle-size and high-concentration aggregates, the low-concentration particle solution will also partially enter the low-permeability section to play a role in profile control and flooding. It can be seen that the ability of particles to migrate to the low-permeability section to perform profile control is not only dependent on the particle size but also on the concentration of the particles.

#### 2.2.4. Analysis of Enhanced Oil Recovery (EOR) under Different Parameters

By using image analysis software to quantitatively analyze the remaining oil in each stage of the model. Based on the results presented in Table 2, it is evident that when altering the heterogeneous distribution form and injection conditions, the enhanced oil recovery (EOR) during the particle flooding stage remains predominantly below 0.5%. The overall enhanced oil recovery (EOR) is mainly influenced by the subsequent water drive flow and the expansion and migration of particles. This indicates that the effect of adjustment is greater than that of flooding. On the other hand, Yuan Chengdong believes that enhancing the microscopic oil displacement efficiency is typically accomplished by increasing the capillary number. The lower the interfacial tension between the displacing phase and the displaced phase, the higher the capillary number [38]. According to the definition of the Newtonian capillary number, along with the interfacial tension mentioned above, the viscosity of the displacing phase, the velocity of displacement, and the contact angle, the capillary number can be calculated to be approximately on the order of 10^−3^ [39]. Therefore, the capillary number of the gel particle agent is smaller and the oil washing efficiency is poor.

The EOR in Experiment 1 is the highest. Compared with Experiment 2, the 3.4% increase in EOR indicates that the gel particles have a better effect on the vertical heterogeneity. The difference in enhanced oil recovery (EOR) between Experiment 1 and Experiment 3, utilizing pellet flooding followed by subsequent water flooding, is 1.98%. Meanwhile, the EOR difference between Experiment 3 and Experiment 4, employing particle flooding along with subsequent water flooding, is 2.35%. Upon comparing these differences, it becomes evident that the impact of concentration parameters on enhanced oil recovery outweighs that of particle size parameters. Comparing the EOR of particle flooding + subsequent water flooding with different particle sizes and concentrations, it can be observed that the profile control effect of 10 wt% submicron particles is the most effective.

### 2.3. Evaluation of Flooding Effect of Gel Particle Macro Core

Since the experimental conditions of the microscopic model involve normal temperature and pressure, they do not simulate the complex high-temperature and high-pressure environment in the actual reservoir. As a result, the enhanced oil recovery (EOR) is higher than that of the core flooding experiment. Due to the small pore volume of the model, accurately and quantitatively describing the change characteristics of the oil recovery factor and water content in the displacement process is challenging. Therefore, the actual production profile control and displacement effect of the gel particle should be based on the core displacement experiment.

Consistent with the design of micro-scale experimental parameters, the core displacement experiment compares the effects of various particle sizes and concentrations under the conditions of vertical heterogeneity. The results are shown in Table 3. Under similar flooding conditions, the final enhanced oil recovery of submicron particles is higher than that of micron particles. In the case of particles of the same size, the final enhanced oil recovery of the high-concentration 10 wt% particle solution is greater than that of the low-concentration 5 wt% particle solution, which aligns with the enhanced oil recovery (EOR) analysis of the microscopic model.

**Figure 10:**
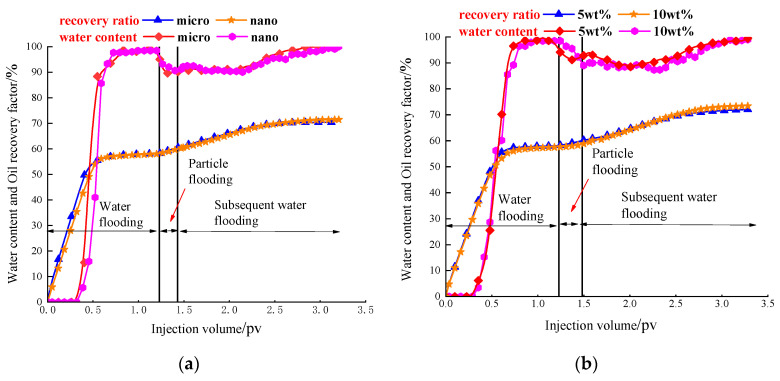
Comparison of the change in the oil recovery factor and water content in the core flooding experiment:(**a**) Change particle size; (**b**) Change particle concentration.

Analysis of the production characteristic curve of the gel particle profile control (Figure 10) shows that before the injection amount reaches about 0.3 pore volumes (pv) in the initial stage, it is necessary to first go through the anhydrous oil production period. During this period, all the produced liquid is oil, and the water content is almost zero. The water content of the produced liquid sharply increases to about 90% after the subsequent injection of approximately 0.2 pore volumes (pv) water. When the water content of the produced fluid reaches about 98%, the efficiency of enhanced oil recovery (EOR) is almost 0%, and it can be considered that the dominant channel of water flooding has been stably formed. The decrease in water content and the increase in the oil recovery factor during the gel particle flooding stage are not significant. This is because the gel particles first infiltrate into the core through the dominant channel, carried by high-salinity water initially. The remaining oil is not utilized during this stage, aligning with the aforementioned microscopic experiment. However, the gel particles are a type of particle agent with a specific diameter. As the injection amount increases, they tend to cause blockages, leading to a preference for flowing through narrower channels during particle flooding. This process helps to drive out the remaining oil. At this time, the water content will decrease and the oil recovery factor will increase gradually. When the particles are heated and expanded in the core, they exert a strong plugging effect on the dominant channel, leading to increased pressure and flow around the smaller pore throat during subsequent water flooding. Based on the pressure data values presented in Table 3, after the gel particles are kept warm for 72 h for subsequent water flooding, the maximum increase in subsequent water flooding pressure can reach 0.24 MPa. This suggests that the expanded particles exhibit a more effective plugging effect on larger pores. At present, the gel particles have been applied in Well Group H, Block T, Tahe Oilfield, and have produced a noticeable profile control effect. The oil production has increased to a peak of 26.4 t/d, and the comprehensive water cut has decreased to a minimum of 32.1%.

## 3. Conclusions

(1) From the results of primary water flooding, the formation law of water flooding channels is determined by the heterogeneity distribution of the model and the connection between different permeability sections. There are five types of remaining oil after primary water flooding: contiguous block, multi-branch cluster, parallel throat, corner concave, and dispersed solitary drop, mainly focusing on the first three types;
(2) The profile control effect of gel particles on reservoir vertical heterogeneity is better than that on reservoir lateral heterogeneity. The 10 wt% submicron particle (600 nm) can penetrate into the channel with a designed throat radius of 5–10 μm in the low-permeability section and the medium−high-permeability section to effectively plug it. The 10 wt% micron-sized particles (2.63 μm) can penetrate the channel with a designed throat radius of 20–30 μm in the high-permeability section to effectively plug it. However, it is nearly impossible for them to penetrate and cause plugging in sections with medium and low permeability. The 5 wt% micron-sized particles (2.63 μm) can penetrate into the channel with a designed throat radius of 10–20 μm in the medium-permeability section and high-permeability section, mainly forming weak plugging in the medium- and high-permeability section;
(3) Both microscopic experiments and macroscopic core displacement experiments show that 10 wt% submicron particles have the best effect on profile control and flooding. The concentration parameters have a more significant impact on the effectiveness of profile control and flooding. The mechanism of action of gel particles is primarily characterized by the agent initially entering the dominant channel and subsequently expanding under the influence of high-temperature water. This process results in the phenomenon of plugging, migration and deformation, and re-plugging. Therefore, it has an efficient profile control and flooding effect on heterogeneous reservoirs.

The effects of various key parameters of the expanded gel particles were investigated using an indoor physical model. Since the model could not simulate the actual conditions of the high-temperature and high-salt reservoir, as well as the changes in the pore throat after long-term water flooding, the experimental discussion may be inadequate. In the future, more attention should be paid to the similarity between the model and the actual field conditions so that the conclusions of laboratory experiments can better guide the field application. In addition, considering that the oil washing efficiency of the gel particle agent is not high, the use of nano-sized particles with smaller particle sizes or with surfactants can be considered to enhance collaborative oil flooding.

## 4. Materials and Methods

### 4.1. Visual Microscopic Glass Etching Experiment

**Figure 11:**
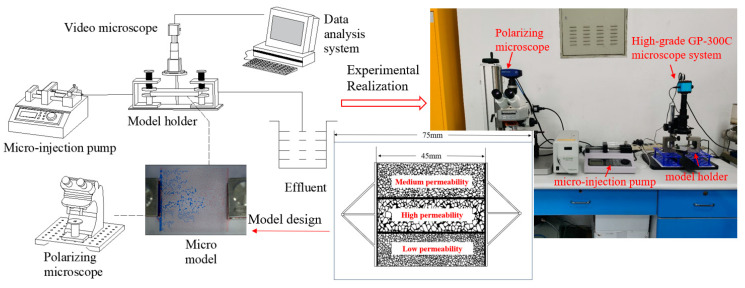
Visualization of microscopic model displacement experiment.

Using the visual microscopic glass etching model, we designed various key parameters for particles. The aim was to explore the mechanism of profiling and flooding effects caused by the key parameters of the gel particle profile control agent at the micro level. Furthermore, we studied the mechanism of water flooding and the distribution of remaining oil. Equipment: high-grade GP−300C microscope system, polarizing microscope, micro-injection pump, model holder, etc., as shown in Figure 11. The internal pore size of the microscopic model is 45 mm × 45 mm. The penetration zone and three connected channels are etched at the inlet and outlet of the model. The upper, middle, and lower parts of the three penetration sections correspond to different permeability zones: the medium-permeability zone with a throat diameter of 10–20 μm, the high-permeability zone with a throat diameter of 20–30 μm, and the low-permeability zone with a throat diameter of 5–10 μm.

The simulated oil was a mixture of white oil and aviation kerosene in proportion to maintain a viscosity ratio of approximately 5.8:1 at room temperature. It was dyed red with Sudan III and filtered to prevent undissolved dyes from blocking the throat. The simulated water salinity was 210,000 mg/L, and the injected water was stained with methyl blue and then filtered. The gel particle profile control agent is a stable particle system formed by a high viscoelastic polymer gel through high-speed mechanical shear. The gel particle profile control agents used in this paper are composed of submicron particles with an initial median diameter of 600 nm and micron particles with an initial median diameter of 2.63 μm. Under the actual high-temperature and high-salt conditions of the reservoir (102 °C, 210,000 mg/L), the gel particle size can expand approximately four times within 72 h. Moreover, the viscosity of 10 wt% gel particles can be sustained at around 3 mPa·s after shear for 5, 10, 15, 20, and 30 min at 1000 rpm, respectively. At normal temperature, the interfacial tension of oil and water is 25.6 mN/m. This tension decreases by 2.3 mN/m and 1.2 mN/m, respectively, after the addition of submicron and micron profile control agents. By utilizing the sand-filled tube model, the system’s plugging rate can remain above 80% after 30 days of aging, even under the storage environment of high temperature and high salt. The wettability of the model is hydrophilic, and the contact angle of the injected water in the model is 39.89°.

Experimental steps:

1. The software was connected to the microscope and the parameters adjusted;
2. The vacuum-saturated simulation of oil was modeled at room temperature;
3. The simulated water was injected at a constant speed of 50 μL/min until the pressure stabilized, and the injection volume reached approximately 320 μL;
4. According to the experimental scheme in Table 4, various particle sizes and concentrations of profile control and flooding agents were injected. Subsequently, the formation temperature was maintained for 72 h;
5. Subsequent water flooding. The experiment was carried out at room temperature, and the injection pressure, injection volume, and injection speed were recorded. The displacement process and local feature images were taken.

### 4.2. Heterogeneous Rectangular Core Displacement Experiment

**Figure 12:**
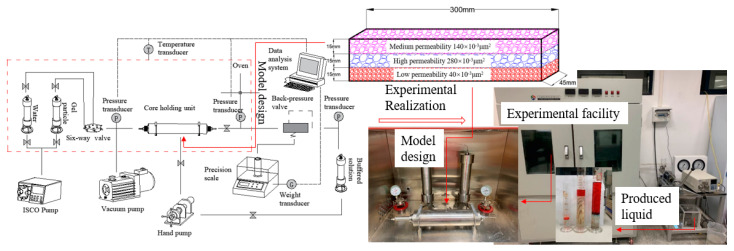
Heterogeneous three-layer long core displacement experiment.

It is used to analyze the profile control and flooding effects of gel particles and verify the characteristics of microscopic profile control and flooding mechanisms. Equipment: High-temperature and high-pressure core flow evaluation device includes an advection pump, thermostat, hand pump, core holder, intermediate container, etc., as shown in Figure 12. The simulated water and gel particle profile control agent is the same as that used in the microscopic experiment. The simulated oil is industrial white oil (102 °C, 1.64 mPa·s). The permeability design of the three-layer heterogeneous core model was based on the permeability distribution of the perforated interval of the control and flooding well group selected in the T block of the Tahe oilfield. Finally, the permeability design for the rectangular three-layer heterogeneous core used in the laboratory experiment was upper/middle/lower corresponding to the medium permeability/high permeability/low permeability = 140/280/40 × 10^−3^ μm^2^, 30 × 4.5 × 4.5 cm.

Experimental steps:

1. The air tightness of the equipment was tested;
2. Core drying, weighing, and vacuum pressure saturation simulation of the oil was carried out;
3. Formation temperature aging for 24 h was conducted;
4. Water flooding of the core was stopped when the water content reached 98% at the outlet end;
5. According to the experimental scheme, the flooding agent was injected and maintained at the formation temperature for 72 h;
6. Subsequent water flooding to the outlet end of the water content reached 98%. The experimental formation temperature was carried out, the pressure, produced water, and oil were recorded, and the change trend in water content and the oil recovery factor were then calculated.

By comparing the permeability measured by the gas and liquid of each core, it can be seen from Table 5 that the gas permeability and liquid permeability measured by different cores show little difference, which accords with the experimental error range. The experiment completed by using this model is credible.

## Figures and Tables

**Table 1:** The pressure performance at different displacement stages under different parameters.

Number	Placement Mode	Injection Particle Sized	Injection Concentration/wt%	Injection Volume/μL	Water Flooding Pressure /MPa	Particle Flooding Pressure /MPa	Subsequent Water Flooding Pressure /MPa
1	vertical	submicro	10	100	0.005	0.006	0.016
2	horizontal	submicro	10		0.003	0.004	0.011
3	vertical	micron	10		0.0035	0.0075	0.0165
4	vertical	micron	5		0.0075	0.0075	0.0175

**Table 2:** The EOR performance at different stages under different parameters.

Number	Experimental Parameters	Oil Recovery/%			Enhanced Oil Recovery (EOR)/%		
		Water Flooding	Particle Flooding	Subsequent Water Flooding	Particle Flooding	Subsequent Water Flooding	Particle + Subsequent Water Flooding
1	vertical, 10 wt% submicron	55.98	56.38	78.30	0.40	21.92	22.32
2	horizontal, 10 wt% submicron	59.43	59.84	78.36	0.41	18.51	18.92
3	vertical, 10 wt% micron	57.86	58.28	78.2	0.42	19.92	20.34
4	vertical, 5 wt% micron	56.28	56.6	74.27	0.32	17.67	17.99

**Table 3:** The pressure and oil recovery at different stages under different parameters.

Condition	Parameters	Pressure/MPa			Oil Recovery/%			Ultimate Oil Recovery/%	EOR/%
		Water Flooding	Particle Flooding	Subsequent Water Flooding	Water Flooding	Particle Flooding	Subsequent Water Flooding		
Vertical heterogeneity, similar amount of profile control flooding	Micro particles	0.06	0.31	0.45	58.25	2.26	9.86	70.37	12.12
	Submicro particles	0.08	0.30	0.46	57.98	2.51	10.95	71.44	13.46
Vertical heterogeneity, submicron particles	5 wt%	0.07	0.31	0.55	58.02	2.78	11.25	71.44	14.03
	10 wt%	0.08	0.33	0.57	57.61	2.88	13.01	73.50	15.89

**Table 4:** Visualization microscopic experiment scheme.

Number	Placement Mode	Injection Particle Type	Injection Concentration/wt%	Injection Volume/μL
1	vertical	submicron	10	100
2	horizontal	submicron	10	
3	vertical	micron	10	
4	vertical	micron	5	

**Table 5:** Basic parameters of the core used in the experiment.

Condition	Parameters	Gas Permeability/10^−3^ μm^2^	Liquid Permeability/10^−3^ μm^2^	Porosity/%
Vertical heterogeneity, similar amount of profile control flooding	micro particles	154	140.55	20.44
	submicron particles	156	141.98	20.58
Vertical heterogeneity, submicron particles	5 wt%	156	140.36	20.24
	10 wt%	152	140.45	20.96

## Data Availability

The data presented in this study are openly available in the article.
